# Clinical Presentation and Pathogenesis of Cold-Induced Autoinflammatory Disease in a Family With Recurrence of an *NLRP12* Mutation

**DOI:** 10.1002/art.30170

**Published:** 2011-03

**Authors:** S Borghini, S Tassi, S Chiesa, F Caroli, S Carta, R Caorsi, M Fiore, L Delfino, D Lasigliè, C Ferraris, E Traggiai, M Di Duca, G Santamaria, A D'Osualdo, M Tosca, A Martini, I Ceccherini, A Rubartelli, M Gattorno

**Affiliations:** 1 Istituto G. Gaslini and University of GenoaGenoa, Italy; 2 National Cancer Research InstituteGenoa, Italy; 3 Istituto G. GasliniGenoa, Italy; 4 Sanford-Burnham Medical Research InstituteLa Jolla, California

## Abstract

**Objective:**

*NLRP12* mutations have been described in patients affected with peculiar autoinflammatory symptoms. This study was undertaken to characterize *NLRP12* mutations in patients with autoinflammatory syndromes, particularly a novel missense mutation, p.D294E, affecting a protein sequence crucial for ATP binding, which was identified in a Caucasian family with familial cold-induced autoinflammatory syndrome in some family members.

**Methods:**

Fifty patients were tested for *NLRP12* mutations. A Caucasian family with the p.D294E missense mutation of *NLRP12* in some family members was clinically characterized. In vitro analysis of the effects of the mutation on NF-κB activity was performed in HEK 293 cells after cotransfection of the cells with a luciferase NF-κB–responsive element and mutant or wild-type (WT) *NLRP12* expression plasmids. NF-κB activity was also evaluated 24 hours after stimulation with tumor necrosis factor α in monocytes from individual family members carrying the mutation. Furthermore, secretion of interleukin-1β (IL-1β), production of reactive oxygen species (ROS), and activation of antioxidant systems in patient and healthy donor monocytes, under resting conditions and after stimulation with pathogen-associated molecular patterns (PAMPs), were also assessed.

**Results:**

In the family assessed, the p.D294E mutation segregated in association with a particular sensitivity to cold exposure (especially arthralgias and myalgia), but not always with an inflammatory phenotype (e.g., urticarial rash or fever). In vitro, the mutant protein maintained the same inhibitory activity as that shown by WT *NLRP12*. Consistently, *NLRP12*-mutated monocytes showed neither increased levels of p65-induced NF-κB activity nor higher secretion of IL-1β. However, the kinetics of PAMP-induced IL-1β secretion were significantly accelerated, and high production of ROS and up-regulation of antioxidant systems were demonstrated.

**Conclusion:**

Even with a variable range of associated manifestations, the extreme sensitivity to cold represents the main clinical hallmark in an individual carrying the p.D294E mutation of the *NLRP12* gene. Although regulation of NF-κB activity is not affected in patients, redox alterations and accelerated secretion of IL-1β are associated with this mild autoinflammatory phenotype.

Autoinflammatory syndromes are a group of diseases characterized by recurrent episodes of systemic inflammation that are accompanied by fever and often associated with other clinical manifestations, such as rash, serositis (peritonitis, pleuritis), lymphadenopathy, arthritis, and systemic reactive amyloidosis as severe long-term complications (1). Among the autoinflammatory syndromes, cryopyrin-associated periodic syndromes (CAPS) are conditions associated with mutations of the *NLRP3* gene, also known as *CIAS1* or *PYPAF1*, a key component of the inflammasome directly involved in the mechanism of interleukin-1β (IL-1β) processing and secretion. CAPS include familial cold-induced autoinflammatory syndrome (FCAS), Muckle-Wells syndrome (MWS), and chronic infantile neurologic, cutaneous, articular syndrome (CINCA syndrome) (2,3). These disorders are associated with gain-of-function *NLRP3* heterozygous mutations, which determine an abnormal in vitro secretion of IL-1β by monocytes (4). The specific inhibition of IL-1β leads to a dramatic normalization of the inflammatory manifestations (5,6) as well as a strong reduction in IL-1β secretion (7).

In addition, heterogeneous clinical manifestations have been observed in several families with CAPS (8), suggesting that modifier genes and/or environmental factors are involved in the disease phenotype. Patients with clinical manifestations attributable to CAPS but without mutations at the *NLRP3* locus have been described and, in a subset of these *NLRP3*-negative patients, mutations in the *NLRP12* gene, also known as *MONARCH-1* or *PYPAF7*, have recently been identified (9).

The nucleotide binding domain leucine-rich repeat containing protein (NLRP) family, also known as CATERPILLER (caspase activation and recruitment domain, transcription enhancer, purine binding, pyrin, lots of leucine repeats), is structurally characterized by a tripartite architecture containing a central nucleotide binding domain (NACHT or NOD), which exhibits ATPase activity and regulates oligomerization, a specific N-terminal effector domain for recruiting downstream effector molecules, and a C-terminal portion consisting of a variable series of leucine-rich repeats implicated in mediating autoregulation, protein–protein interactions, and/or pathogen-associated molecular pattern (PAMP) sensing (10). In humans, at least 22 NLRP family members have been found to play a central role in the regulation of inflammatory and cell death responses, during infections and in autoinflammatory disorders (10). In particular, NLRP-12 acts as a negative regulator of inflammation by suppressing both canonical and noncanonical NF-κB activation and subsequent production of proinflammatory cytokines and chemokines (11,12). In the present study, we describe an Italian family, some of whose members had clinical features consistent with FCAS, in whom a missense mutation of the *NLRP12* gene was found.

## PATIENTS AND METHODS

### Patients

A total of 50 patients who were negative for mutations of *NLRP3* were analyzed for mutations of *NLRP12*. The patients screened in the *NLRP12* mutational search included 2 patients with a history of cold-induced urticarial rash associated with inflammatory features (i.e., fever, arthralgias, and elevation of acute-phase reactants), 5 patients with a clinical phenotype strongly consistent with MWS or CINCA syndrome, including responsiveness to anti–IL-1 treatment, 11 patients who had already been screened for genes associated with periodic fever (MEFV, MVK, and TNFRSF1A) and had presented with at least 1 of the typical manifestations during fever attacks (urticarial rash, hearing loss, and sensitivity to cold), 12 patients with systemic-onset juvenile idiopathic arthritis displaying a complete response to anti–IL-1 treatment (13–15), and 20 patients with a history of cold-induced urticaria without evidence of features of inflammation.

Eighty healthy individuals of Italian origin, randomly selected among blood donors at the Gaslini Institute, were analyzed as healthy control subjects, after providing their agreement on a proper consent form. The present study was approved by the Gaslini Institute ethics committee (Genoa, Italy).

### Mutation screening

The sequence of the sole exon 3 of the *NLRP12* gene was analyzed for mutations by polymerase chain reaction (PCR) (Table 1), followed by direct sequencing using BIG DYE (version 1.1; Applied Biosystems) and an Applied Biosystems 3130 automated sequencer.

**Table 1:** Conditions of the polymerase chain reaction (PCR)

			PCR conditions*	
			
Fragment	PCR size, bp	Primer	Protocol†	Enhancer
Fragment A	643	Forward 5′-GAGTAGCTGGAACTACAGGC-3′, reverse 5′-GCTCATCGAAGCCGTCGATG-3′	94°C for 30 minutes, 62°C for 40 minutes, 72°C for 40 minutes (28 cycles)	
Fragment B	497	Forward 5′-GAAGCTCTTCCAAGGCAGATT-3′, reverse 5′-CGAAGCACATGGTGAAGAGA-3′	94°C for 30 minutes, 60°C for 40 minutes, 72°C for 40 minutes (30 cycles)	5% DMSO
Fragment C	499	Forward 5′-TCTGAGGCAGAAAGGAAGGA-3′, reverse 5′-CCCCCTCGTCCAGGATATAG-3′	94°C for 30 minutes, 55°C for 40 minutes, 72°C for 40 minutes (35 cycles)	5% DMSO
Fragment D	441	Forward 5′-TTCCAGAAGGACATCAACTGTG-3′, reverse 5′-CTCCATCTTGGAGGCAATGT-3′	94°C for 30 minutes, 55°C for 40 minutes, 72°C for 40 minutes (35 cycles)	5% DMSO
Fragment E	274	Forward 5′-CAGCTGCTTGTACGAGATCC-3′, reverse 5′-CACGAACCTAAGCAGCCCCA-3′	94°C for 30 minutes, 60°C for 40 minutes, 72°C for 40 minutes (30 cycles)	

* One hundred nanograms of DNA was amplified in a 25-μl reaction containing 10 m*M* Tris HCl, pH 8.3, 1.5 m*M* MgCl_2_, 50 m*M* KCl, 200 μl dNTPs, and 1.25 units *Taq* polymerase (TaqGold; Applied Biosystems) with 1 μ*M* of each appropriate PCR primer.

† An initial denaturation step at 95°C for 10 minutes and an extension cycle at 72°C for 7 minutes at the end were performed.

### Constructs and transfections

The wild-type (WT) sequences of *NLRP12* complementary DNA (cDNA) were obtained by PCR from a MGC commercial clone and inserted into the pcDNA3.1 vector. Mutant *NLRP12* constructs were generated by site-directed mutagenesis starting from this same construct, as described previously (16), using the oligonucleotides 5′-TCATCATCGAGGGCTTCGATG-3′ and 5′-CATCGAAGCCCTCGATGATGA-3′ to introduce the p.Asp294Glu substitution.

Plating of HEK 293 cells (2 × 10^5^) on 24-well plates was performed at the same time as the transfection, using Fugene 6 (Roche), following the manufacturer's instructions. NF-κB activity was tested using a synthetic promoter from Stratagene, consisting of several repetitions of the canonical NF-κB binding site, cloned upstream of the reporter luciferase cDNA. Fifty nanograms of this construct and 5 ng of *Renilla* luciferase reporter plasmid pRL-CMV (Promega), used as a transfection efficiency control, were cotransfected together with the expression vectors. In particular, 100 ng of IL-1 receptor–associated kinase 1 (IRAK-1) or myeloid differentiation factor 88 (MyD88) expression vectors (a kind gift from Dr. J. Tschopp, Lausanne, Switzerland), were used for induction of NF-κB activity and were cotransfected with 150 ng of either the *NLRP12* plasmid or pcDNA3.1 empty vector. Each experiment was performed in triplicate and repeated at least 3 times.

### NF-κB activation assay

NF-κB activation was evaluated on nuclear extracts of monocytes prepared from the peripheral blood of healthy donors and patients. Monocytes were treated with 10 ng/ml tumor necrosis factor α (TNFα; R&D Systems) for 24 hours or left untreated. The TransAM NF-κB p65 kit (Active Motif) was used to assess possible NF-κB activation, with the primary NF-κB p65 antibody recognizing an epitope on p65 that becomes accessible only when NK-κB is activated and bound to its target DNA. The anti-rabbit horseradish peroxidase–conjugated antibody that was used as secondary antibody provides a sensitive colorimetric readout that is easily quantified by spectrophotometry. The adsorbance was read within 5 minutes at 450 nm, with an optical reference wavelength of 655 nm. Raji nuclear extracts (Active Motif) were provided as a positive control for NF-κB activation.

### Pattern of IL-1β secretion

Blood samples were obtained from patients with the *NLRP12* mutation and from 10 age-matched healthy control subjects, after informed consent was provided. Monocytes from the freshly withdrawn heparinized blood samples were enriched by adherence (7,13).

The monocytes were then incubated in RPMI 1640 containing 5% fetal bovine serum (FBS) (Sigma-Aldrich) and activated with 1 μg/ml lipopolysaccharide (LPS), 3 μg/ml muramyl dipeptide, or 50 μg/ml of the yeast cell wall derivative zymosan (all from Sigma-Aldrich), at 37°C for different lengths of time (7,13). Supernatants were collected for determination of IL-1β secretion. The amount of IL-1β secretion in the monocyte supernatants was determined in triplicate, by enzyme-linked immunosorbent assay (R&D Systems) (13).

### Determination of intracellular reactive oxygen species (ROS)

Monocytes were stimulated with LPS, and 10 μ*M* of dichlorofluorescein diacetate (H_2_DCF-DA; Molecular Probes) was added to the cultures 30 minutes before the end of the incubation. Fluorescence was measured in cell lysates with a microplate fluorimeter, with excitation at 480 nm and emission at 530 nm for H_2_DCF-DA. Data were normalized to the protein content of cell lysates, as measured by the Lowry method (17). Duplicate experiments were performed for each sample.

### Determination of cysteine in culture medium

Supernatants (0.1 ml) from monocytes cultured at 4 × 10^5^/0.5 ml in 24-well plates in RPMI–5% FBS were reacted with 10 m*M* 5,5′-dithiobis-(2-nitrobenzoic acid) (Sigma-Aldrich), and the absorption was measured at 412 nm. Cysteine (Sigma-Aldrich) was used as standard (17). Triplicate experiments were performed for each sample.

### Real-time PCR

Total RNA was isolated from monocytes using TriPure Isolation Reagent (Roche) and reverse-transcribed using Superscript III Reverse Transcriptase (Invitrogen). Real-time PCR determination of cystine transporter (xCT) and thioredoxin cDNA was performed as described previously (15), using SYBR Green ER quantitative PCR Supermix for the iCycler Reagent (Invitrogen). The specific primers used were as follows: forward 5′-AAACCCAAGTGGTTCAGACG-3′ and reverse 5′-ATCTCAATCCTGGGCAGATG-3′ (for xCT), forward 5′-AGCAGATCGAGAGCAAGACT-3′ and reverse 5′-CACTCTGAAGCAACATCCTG-3′ (for thioredoxin), and forward 5′-ATGGCCTTCCGTGTTCCTAC-3′ and reverse 5′-GCTTCACCACCTTCTTGATGTC-3′ (for GAPDH). Relative expression of messenger RNA (mRNA) was determined using the ΔC_t_ method.

### Statistical analysis

The data were statistically analyzed by one-way analysis of variance, followed by posttest Bonferroni correction, or by unpaired *t*-test using GraphPad software. Differences were considered statistically significant at *P* values less than or equal to 0.05.

## RESULTS

### *NLRP12* mutation screening and clinical features of patients carrying a coding variant

Among a large panel of individuals affected with autoinflammatory disorders, 50 patients were selected for molecular screening of the *NLRP12* gene. Since a previous study identified mutations exclusively in the third exon of the gene (9) and, similarly, mutations in different regions of the *NLRP3* gene, belonging to the same CATERPILLER family, have been detected only occasionally (18), we decided to limit the mutation screening of *NLRP12* solely to exon 3. A number of common variants were detected, in addition to 1 missense heterozygous nucleotide change, c.882C>G, leading to the amino acid substitution p.Asp294Glu. By performing software simulations with the online software programs Poliphen and SIFT (available at http://genetics.bwh.harvard.edu/pph/ and http://blocks.fhcrc.org/sift/SIFT.html, respectively), we predicted a deleterious effect of this mutation, which turned out to be located in the highly conserved sequence Walker B, considered necessary for ATP binding and for subsequent activity in the protein (19).

The individual who was found to be a carrier of the p.Asp294Glu mutation, a 32-year-old woman, came to our observation for the first time in January 2006 with a suspected diagnosis of FCAS. Since the age of 20 years, she had experienced recurrent episodes of urticarial rash associated with fever, arthralgias, myalgia, and headache. The clinical manifestations occurred exclusively during winter time and were exacerbated by cold exposure. The rash was generally itchy and involved the face, arms, and trunk. Fever episodes were associated with elevations in the levels of acute-phase reactants, with normalization of levels during symptom-free intervals. Episodes occurred for an overall duration of 7–15 days and showed a clinical response to steroid and antihistamine administration. The patient was found to be negative for antinuclear antibodies, antineutrophil cytoplasmic antibodies, and complement consumption. The skin-ice test and test for allergies consistently yielded negative results. The patient displayed complete well-being during the warm season. Prophylactic administration of low-dose steroids and antihistamines during the winter time resulted in prevention of the clinical manifestations. No hearing loss or ocular involvement (papilledema, uveitis) were observed.

**Figure 1:**
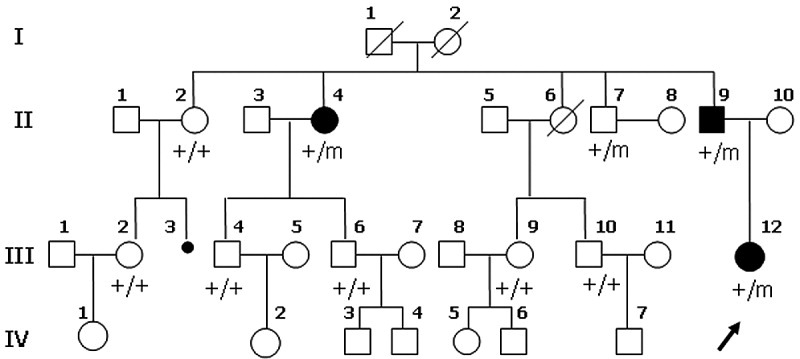
Genealogy tree of the Italian Caucasian family identified as having a recurrence of the p.D294E substitution (represented by “m”) in the *NLRP12* gene. Each symbol represents an individual female (circle) or male (square) family member, with Roman numerals indicating the generational level. **Arrow** indicates the proband, large solid symbols indicate individuals with clinical manifestations, small solid symbol indicates abortion, and slashes indicate death.

The p.Asp294Glu mutation was also identified in 3 other family members (Figure 1). The proband's father (patient II-9 in Figure 1), a 61-year-old otherwise-healthy man, reported having a clear sensitivity to cold exposure since childhood, with arthralgias and myalgia variably associated with a mild and transient urticarial rash. These episodes were generally well tolerated and did not significantly influence his daily activities. He was treated at demand with nonsteroidal antiinflammatory drugs (NSAIDs) to control the symptoms. Clinical manifestations were generally prevented by avoiding exposure to the cold. In fact, a clear exacerbation of the clinical manifestations was observed after cold exposure (i.e., air conditioning or entry into the frozen food section of the supermarket). No unexplained fever episodes were reported. Whenever tested, the levels of acute-phase reactants were in the normal range. However, due to the modest severity and short duration of his clinical manifestations, the patient was never tested during the presence of cold-induced symptoms. Moreover, a cold-provocation test was not performed, due to the patient's refusal. Results of allergy tests and the skin-ice test were negative. No hearing loss or cutaneous manifestations were present.

A 78-year-old woman, aunt of the proband, was also affected (patient II-4 in Figure 1). Since infancy, she had experienced arthralgias and myalgia, variably associated with an urticarial rash, with limitation of daily activities. Low-grade fever and elevation in the levels of acute-phase reactants were also reported. At the age of 38 years, the patient developed an optic neuritis, which was treated with steroids. Similar to the other affected family members, her clinical manifestations were present during the winter time, with complete well-being during the warm season. Evident sensitivity to cold exposure (“frozen food section” symptoms) was also reported. Over time, a clinical diagnosis of urticarial vasculitis was suspected. The clinical manifestations were partially controlled by NSAIDs and by variable courses of steroids at the time of exacerbation of the clinical manifestations.

The fourth carrier of the p.Asp294Glu *NLRP12* mutation within the family was a 67-year-old man, uncle of the proband (patient II-7 in Figure 1). At variance with the other affected family members, he denied ever having any evident clinical manifestations associated with cold exposure. Conversely, an urticarial-like rash was observed, in conjunction with episodes of fever, which was, according to the patient's judgment, mainly associated with intercurrent viral or bacterial infections. No musculoskeletal manifestations, hearing loss, or ocular manifestations were reported. The patient died of myocardial infarction soon after the identification of the molecular defect. Another 6 asymptomatic members of the family were screened and were found to carry WT *NLRP12*.

### In vitro analysis of the *NLRP12* mutation effect on NF-κB activity

In the original report describing, for the first time, patients affected with *NLRP12*-related periodic fever, in vitro analysis of transfected cells showed that the mutations found were associated with an impairment of the *NLRP12*-mediated inhibitor activity on the NF-κB pathway (9). In particular, the truncated p.Arg284X and the frameshift Val635ThrfsX12 *NLRP12* mutations were demonstrated to be functionally associated with high levels of NF-κB activity, thus accounting for the autoinflammatory phenotype. We therefore decided to apply the same approach, based on the cotransfection of a luciferase NF-κB promoter reporter construct with either mutant or WT *NLRP12* expression plasmids in HEK 293 cells, to test the p.Asp294Glu *NLRP12* mutation.

**Figure 2:**
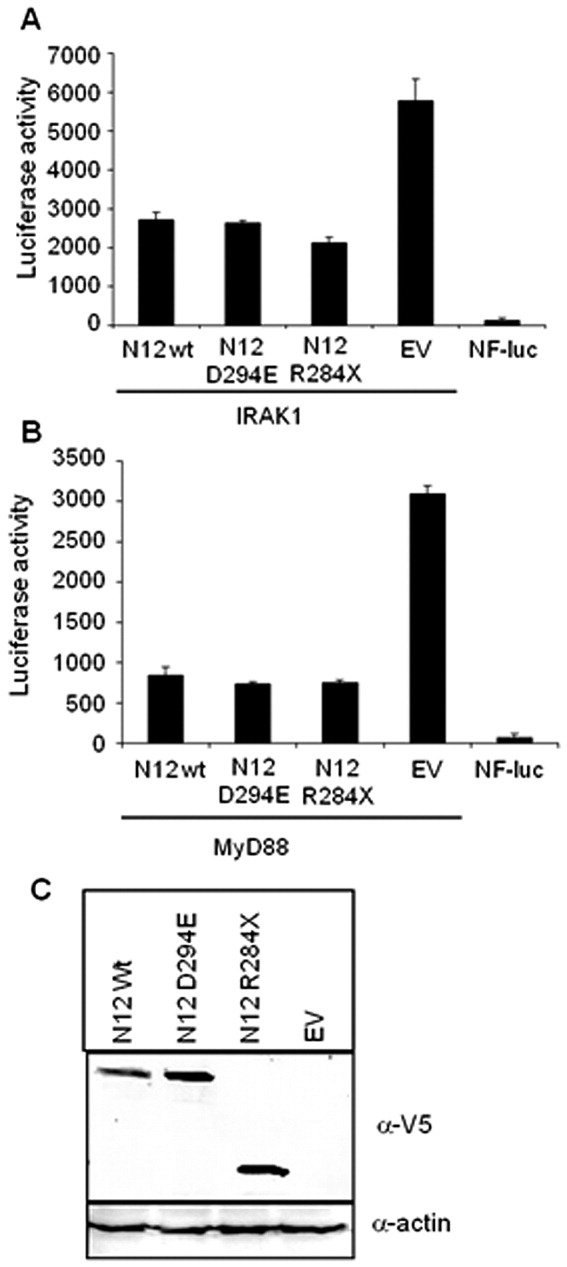
Results of luciferase assays in HEK 293 cells, testing the inhibitory capability of wild-type (WT) and mutant *NLRP12* (N12) proteins on NF-κB signaling. **A** and **B**, Interleukin-1 receptor–associated kinase 1 (IRAK-1) **(A)** or myeloid differentiation factor 88 (MyD88) **(B)** was transfected to induce NF-κB activity. Cotransfections included *NLRP12* WT or mutant (p.D294E missense or p.Arg284X nonsense) variants, pcDNA3.1 empty vector (EV), or a construct containing NF-κB binding sites upstream of the luciferase reporter gene (NF-luc), along with adequate combinations of plasmid as controls. Bars show the mean ± SD of triplicate experiments. **C**, Western blotting of lysates from the same cells as in **A** and **B** was performed to confirm expression of the NLRP-12 proteins cloned with the V5 epitope. The antibody α-actin was used to verify equal amounts of the different lysates.

**Figure 3:**
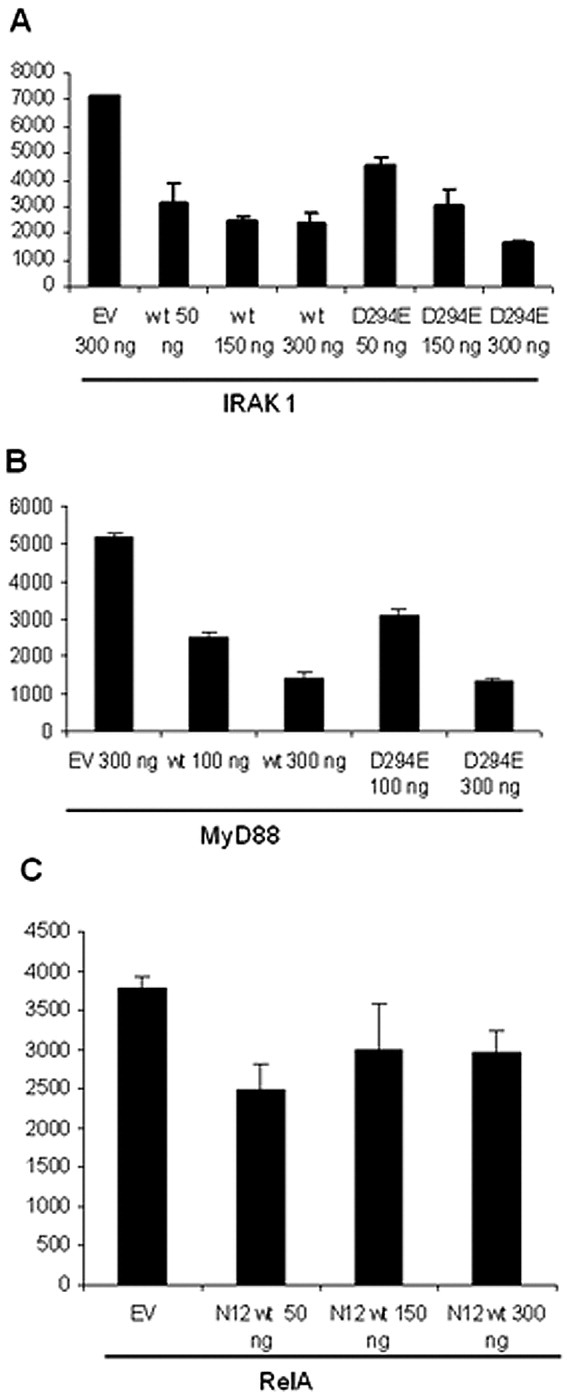
Dose effects of interleukin-1 receptor–associated kinase 1 (IRAK-1) **(A)**, myeloid differentiation factor 88 (MyD88) **(B)**, or p65-RelA **(C)** expression vectors transfected to induce NF-κB activity, as determined in luciferase assays using HEK 293 cells. Increasing amounts of both wild-type (WT) and mutant *NLRP12* (N12) displayed increasing inhibitory effects on NF-κB signaling, as indicated by reductions in the relative luciferase activity (y-axis). Bars show the mean ± SD of triplicate experiments. EV = pcDNA3.1 empty vector.

The NF-κB–dependent luciferase activity induced by overexpression of IRAK-1 or MyD88 was analyzed in cotransfection experiments with either WT or mutant *NLRP12* plasmids (Figures 2A and B). Contradicting the results expected based on the in silico prediction, the p.Asp294Glu-mutated protein maintained the same NF-κB–inhibitory activity as that shown by WT *NLRP12*, even when increasing amounts of the expression plasmids were used (Figures 3A and B). Surprisingly, at variance with previously reported findings (9), the p.Arg284X nonsense mutation was unable to prevent the inhibitory effect of WT *NLRP12*. Moreover, in contrast to the findings already reported (9) but consistent with other previous data (12), we could not observe any inhibitory effect of WT *NLRP12* on NF-κB activity induced by p65 (Figure 3C). For this reason, the p.Asp294Glu mutation was not tested in this experimental design. To rule out the possibility that the absence of effects of the mutated *NLRP12* on NF-κB activity was due to unsuitability of the cell model used (human embryonic kidney cells), the same experiment was repeated in the monocytic cell line THP-1. Even in these cells, however, the same absence of effect was observed (results not shown).

### Ex vivo analysis of NF-κB activity in patients with the *NLRP12* mutation

To further investigate the role of the p.Asp294Glu gene variant in the cold-induced autoinflammatory clinical features recurring in the proband's family, we undertook an analysis of the expression of the active form of NF-κB in primary cells from family members with the mutation. In particular, we analyzed the expression of NF-κB in 3 family members carrying the p.Asp294Glu *NLRP12* mutation, all of whom, at the moment of the study, were asymptomatic.

**Figure 4:**
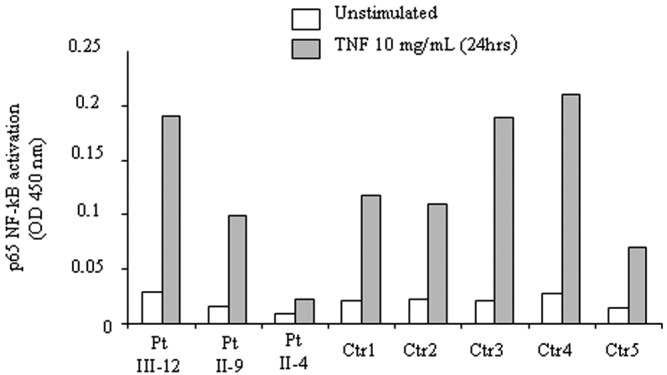
NF-κB activation induced by p65 in monocytes from 3 patients (Pt) with the p.D294E mutation and 5 age-matched healthy controls (Ctr). Monocytes were either left unstimulated or stimulated with 10 ng/ml tumor necrosis factor α (TNFα) for 24 hours. The Active Motif TransAM NF-κB p65 kit was used to assess NF-κB activity. OD = optical density.

Patients with this *NLRP12* mutation did not show increased basal levels of p65-induced NF-κB activity as compared with that in healthy controls (Figure 4). Consistently, when monocytes were stimulated with TNFα, a variable response was observed in both the patients and the healthy controls. In any case, with the exception of patient II-4, whose cells displayed only a modest increment of NF-κB activity after TNFα stimulation, the level of increase in p65-induced NF-κB activity in the other 2 patients with the *NLRP12* mutation was comparable to that observed in healthy controls (Figure 4).

### Patterns of IL-1β secretion, redox state, and stress response in monocytes from patients with the *NLRP12* mutation

**Figure 5:**
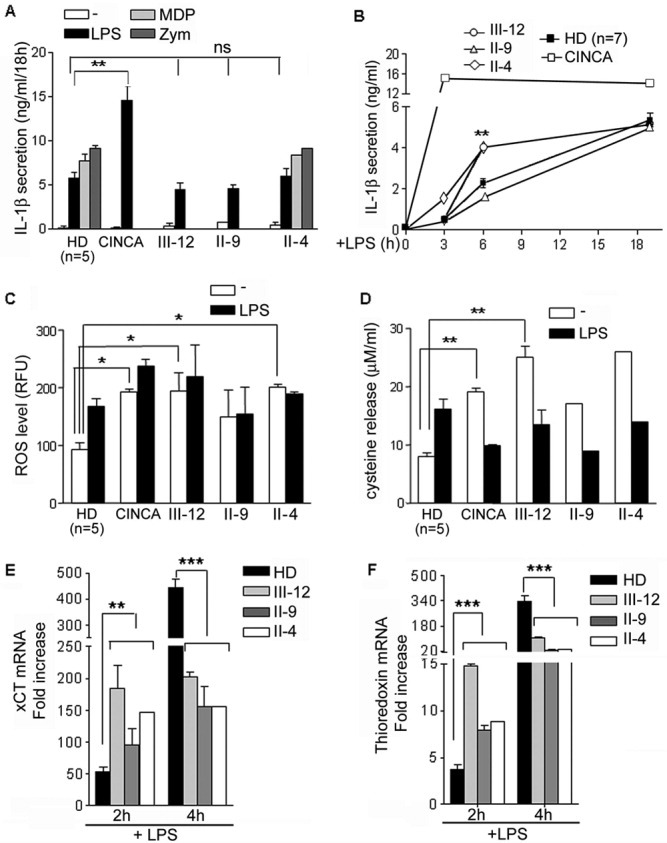
Accelerated kinetics of interleukin-1β (IL-1β) secretion and associated redox alterations in *NLRP12*-mutated monocytes. In these experiments, monocytes from 5–7 healthy donors (HD), a representative patient with chronic infantile neurologic, cutaneous, articular syndrome (CINCA syndrome), and 3 patients with the *NLRP12* mutation were assessed. **A**, Secretion of IL-1β was determined by enzyme-linked immunosorbent assay in monocytes cultured for 18 hours alone or with 1 μg/ml lipopolysaccharide (LPS), 3 μg/ml muramyl dipeptide (MDP), or 50 μg/ml zymosan (Zym). NS = not significant. **B**, The kinetics of IL-1β secretion were assessed after exposure of the monocytes to LPS for 3, 6, or 18 hours. **C**, Intracellular levels of reactive oxygen species (ROS) were determined in monocytes cultured for 1 hour alone or with LPS, with results expressed as relative fluorescence units (RFU). **D**, Extracellular cysteine levels were determined in 18-hour supernatants of monocytes cultured alone or with LPS. Statistical comparisons were not performed for patients II-9 and II-4 due to an inadequate number of experiments. **E** and **F**, Real-time polymerase chain reaction was used to analyze the mean ± SD fold change in levels of cystine transporter (xCT) **(E)** and thioredoxin **(F)** mRNA after 2-hour or 4-hour LPS treatment in monocytes from the 3 patients with the *NLRP12* mutation and 5 healthy donors, relative to that in untreated monocytes. Bars in **A**, **B**, and **D** show the mean ± SD of triplicate experiments; bars in **C** show the mean ± SD of duplicate experiments. ∗ = *P* < 0.05; ∗∗ = *P* < 0.01; ∗∗∗ = *P* < 0.001.

Since *NLRP12* has a close structural homology with *NLRP3*, we investigated whether, as in patients with CAPS, IL-1β hypersecretion in response to Toll-like receptor (TLR) stimulation may also represent the crucial pathogenic event in patients carrying the p.Asp294Glu *NLRP12* mutation. As shown in Figure 5A, *NLRP12*-mutated monocytes from the 3 patients, after stimulation for 18 hours with the TLR-4 ligand LPS, secreted levels of IL-1β that were comparable to those in healthy monocytes, but significantly lower than those secreted by *NLRP3*-mutated monocytes. Triggering of other TLRs by different PAMPs provided similar results.

Interestingly, the kinetics of IL-1β secretion by *NLRP12*-mutated monocytes from patients III-12 and II-4 were accelerated as compared with that in healthy monocytes (Figure 5B); at 6 hours after LPS exposure, the level of secreted IL-1β was more than twice that secreted by controls. A similar, although more dramatic, acceleration of IL-1β secretion, with early achievement of a plateau, was observed in monocytes from a patient with CINCA syndrome (Figure 5B). In contrast, patient II-9, who presented with the mildest clinical manifestations, did not exhibit this pattern.

Redox remodeling is strongly implicated in the process of IL-1β maturation and secretion in healthy conditions (17). TLR triggering induces ROS generation followed by an antioxidant response characterized by up-regulation of the cystine transporter (xCT) and of the oxidoreductase thioredoxin. This up-regulation results in increased uptake of cystine, intracellular conversion to cysteine, and release of reduced cysteine. Interestingly, we have recently shown that the accelerated kinetics of IL-1β secretion by CAPS monocytes is related to redox alterations (20).

Monocytes from patients with CAPS display an unbalanced redox state in resting conditions and an impaired redox response to TLR-mediated oxidative stress, with earlier up-regulation and rapid exhaustion of the antioxidant response. Namely, CAPS monocytes produce more ROS than has been found in control monocytes in basal conditions. The increased level of ROS is responsible for the activation, in resting cells, of antioxidant systems that, after TLR triggering, increase and collapse earlier than in healthy monocytes. This deranged redox response is responsible for the accelerated IL-1β secretion and the earlier achievement of the plateau after LPS stimulation (20).

Thus, we investigated the presence of redox alterations in *NLRP12*-mutated monocytes. As shown in Figure 5C, unstimulated *NLRP12*-mutated monocytes, like CAPS cells, produced more ROS than healthy monocytes. Antioxidant defenses were also activated, as indicated by the higher release of reduced cysteine in the absence of TLR triggering (Figure 5D).

Similar to that in CAPS cells, the antioxidant response to LPS exhausted earlier in *NLRP12*-mutated monocytes. In normal monocytes, up-regulation of the cystine transporter xCT and of thioredoxin was detectable at low levels after 2 hours of LPS exposure and strongly increased after 4 hours. In contrast, in *NLRP12*-mutated monocytes, xCT and thioredoxin mRNA were induced earlier, but the levels increased to a lesser extent compared with that in controls (Figures 5E and F). The rapid exhaustion of the antioxidant systems was confirmed by the decrease in cysteine externalization that was observed following long-term stimulation with LPS (Figure 5D).

Remarkably, monocytes from patient III-12, who had the most severe phenotype and in whom the most accelerated kinetics of IL-1β secretion were observed (Figure 5B), also exhibited the strongest redox derangement, with the highest basal levels of ROS (Figure 5C) and of cysteine release in resting conditions (Figure 5D) and the most rapid up-regulation and exhaustion of thioredoxin and xCT upon stimulation (Figures 5E and F). In contrast, monocytes from patient II-9, who had milder clinical manifestations and in whom the kinetics of IL-1β secretion were comparable to those in controls, exhibited milder alterations of the redox state, although a collapse of the antioxidant response was still observed in this case (Figures 5D–F).

## DISCUSSION

A previous report identified both a nonsense and a frameshift mutation in the sequence of a member of the *NLRP* family, *NLRP12*, in 2 families in whom at least 2 members of the family had a syndrome resembling either FCAS or MWS (9). On the basis of this observation, we selected a number of patients with a clinical phenotype suggestive of CAPS and who were negative for mutations of the *NLRP3* sequence, for *NLRP12* mutation screening. The heterozygous amino acid substitution p.Asp294Glu was identified in a patient affected with a clinical phenotype consistent with a cold-induced autoinflammatory disease, which was found to be absent in a population sample of control chromosomes of the same origin.

As already reported by Jeru et al, the clinical phenotype of the family members other than the probands in their study was rather variable (9). In the family members in our study, the p.Asp294Glu mutation was found to mostly segregate with a particular sensitivity to cold exposure (especially arthralgias and myalgia), even in the absence of urticarial rash, fever, or elevation in the levels of acute-phase reactants. In any case, the clinical manifestations presented by the carriers were generally mild, although quality of life was affected, especially during the winter season in patient II-4 and in the proband. Such phenotypic heterogeneity is frequently observed in autoinflammatory syndromes, as in the case of *NALP3* mutations such as V198M and V200M, which can be associated with CAPS of different severity (18,21). Therefore, we can conclude that wide variability in the expression of the symptoms is a feature of both *NLRP3*- and *NLRP12*-related disorders, presenting phenotypic variability even among members of the same family (8,21).

The missense mutation p.Asp294Glu affects an evolutionarily conserved domain, including that at position 294, which is fundamental for ATP binding and does not allow any change, even a conservative change, in phenotype. This was confirmed in our study not only by the associated disease phenotypes identified in the Caucasian family, but also by the simulations using Polyphen and SIFT software.

To further confirm this finding, we cloned the WT *NLRP12* cDNA and generated the p.Asp294Glu mutant version of the construct, to study in vitro their effect on the NF-κB canonical pathway. In contrast to the findings in the previous study by Jeru et al (9), however, no clear reduction of the inhibitory properties of the p.Asp294Glu mutant of *NLRP12* on NF-κB signaling was observed, as shown by transfection experiments using the IRAK-1 and MyD88 expression vectors. In addition, we could not confirm the decreased inhibitory effect reported for a truncated NLRP-12 protein (9), a discrepancy difficult to explain on the basis of the data obtained under identical experimental conditions, although not with identical constructs.

Finally, we could not test the mutant *NLRP12* effect on p65-induced NF-κB activation. Indeed, in accordance with the findings from a previous biochemical study in which the inhibitory activity of *NLRP12* on NF-κB signaling acted on the initial steps of both the TLR and TNF receptor pathways (12), transfection of *NLRP12* constructs could not inhibit the activity of NF-κB induced by forced expression of p65.

Our experimental approach was designed to investigate the effect of *NLRP12* only on the canonical NF-κB pathway, and this allowed us to exclude such an involvement. However, we cannot exclude the possibility that *NLRP12* has a role in suppressing the noncanonical NF-κB/p100 processing pathway, a mechanism by which the p.Asp294Glu mutation may contribute to the maintenance of high levels of NF-κB activation. This again raises the issue previously discussed by Jeru et al (9), in which the reported results were consistent with a role of not only WT *NLRP12* in the canonical NF-κB pathway but also its mutant *NLRP12*–mediated impairment. In any case, according to Ye et al, an impairment of the ATP binding domain would reduce the antinflammatory activity of *NLRP12* by affecting both the canonical and the noncanonical NF-κB activating pathways (22). This suggests that *NLRP12* may exert its inhibitory effect through a still-unknown, NF-κB–independent pathway.

Our results from in vitro studies were confirmed by the assessment of NF-κB activity levels on patient monocytes, which displayed a response to TNFα stimulation that was comparable to that in healthy control cells. With the aim of identifying alternative pathogenic mechanisms, we therefore analyzed whether *NLRP12* variants could affect the pattern of secretion of IL-1β, as already described in patients with *NLRP3* mutations. The analysis of IL-1β secretion by *NLRP12*-mutated monocytes revealed that, although the global level of the secreted cytokine was comparable to that in healthy monocytes, the kinetics of secretion were accelerated. An accelerated secretion of IL-1β was also observed in CAPS monocytes and was related to a deranged basal redox state and redox response to TLR triggering (20). Interestingly, the same redox signature, characterized by higher basal levels of both ROS and antioxidants and faster up-regulation of the antioxidant systems upon TLR triggering, was shared by monocytes from patients with the *NLRP12* mutation. These data confirm a functional relationship between the kinetics of IL-1β secretion and redox remodeling.

The major implication of our findings is that, whereas in healthy monocytes, IL-1β secretion reaches a plateau after many hours of exposure to PAMPs, when mechanisms aimed at down-modulating IL-1β activity, such as secretion of the IL-1 receptor antagonist (IL-1Ra), are fully operating (23) in *NLRP3*- and *NLRP12*-mutated monocytes, IL-1β reaches the peak of secretion earlier, when IL-1Ra production has not yet started. Thus, a weak TLR stimulation will trigger strong signs of inflammation in patients with the mutations, since IL-1β will be fully secreted soon after TLR engagement, but well before IL-1Ra production.

In patients with CAPS, the concomitance of redox alterations (which accelerate the kinetics of IL-1β secretion) and gain-of-function *NLRP3* mutations (which increase the amount of IL-1β secretion) may act in cooperation to worsen the CAPS phenotype. It is tempting to speculate that in patients carrying *NLRP12* mutations, the mild autoinflammatory phenotype, characterized by weak clinical manifestations, is due to the accelerated kinetics of IL-1β secretion, in the absence of an overall increased amount of the secreted cytokine. The degree of redox alterations would then account for the clinical manifestations of variable entities. Support for this hypothesis comes from the observation that the strongest redox derangement and the fastest secretion of IL-1β were observed in the proband, who displayed the worst clinical signs, whereas monocytes from the father, who presented with a mild phenotype, had milder redox alterations and did not exhibit accelerated IL-1β secretion.

Since neither redox nor the kinetics of secretion are altered in monocytes from patients with nonmonogenic juvenile idiopathic arthritis (20), our results suggest that the chronic derangement of the redox state and response, leading to accelerated IL-1β secretion, is not a common feature of chronic inflammatory diseases, but rather, is a consequence of mutations of *NLRP3* and *NLRP12*. It remains to be seen whether mutations of genes responsible for other autoinflammatory syndromes that respond to IL-1 inhibitors, such as TNF receptor–associated periodic syndrome, pyogenic arthritis, pyoderma gangrenosum, and acne syndrome, and hyperimmunoglobulinemia D with periodic fever syndrome, are also associated with the same redox alterations linked to accelerated IL-1β secretion in monocytes as we observed in patients with *NLRP3* and *NLRP12* mutations.

Thus, the present study showed that the *NLRP12* mutation segregating in an Italian Caucasian family was responsible for the associated mild CAPS-like phenotype, and therefore accounted for a dominant inheritance, characterized by variable expressivity and reduced penetrance. Although the pathogenesis of *NLRP12* mutations has not been completely elucidated, we propose that a deregulation of IL-1β secretion due to an altered redox state and stress response may play a relevant role.
